# Supplementary material for: Relative changes in brain and kidney biomarkers with Exertional Heat Illness during a cool weather marathon
Source: PLoS One. 2022 Feb 17;17(2):e0263873. doi: 10.1371/journal.pone.0263873 (PMC8853487; doi:10.1371/journal.pone.0263873)
Supplement: S1 File — Loc. 1. Treatment facility stationed at 14 mile-point on course (NB course design resulted in runners up to 21 miles being received here). Location II. Main medical tent stationed 100 m behind finishing line. (DOCX) [file pone.0263873.s001.docx]

| Loc. | Case/ Time | | Distance | AVPU | Tc  *°C* | RR  breaths.min^-1^ | HR  beats.min^-1^ | BP  *mmHg* | NSE  *ng.L^-1^* | S100b  *ng.L^-1^* | sCr  *µmol.L^-1^* | cysC  mg.L^-1^ | NGAL  *ug.L-1* | KIM-1  *ng.L^-1^* | Copeptin  *pmol.L^-1^* | CK  *IU.L-1* | TP  *g.L-1* |
| --- | --- | --- | --- | --- | --- | --- | --- | --- | --- | --- | --- | --- | --- | --- | --- | --- | --- |
| I. | 1 | T0  T1 | 14 miles | V  A | 40.1  37.1 | 60  U/D | 115  82 | 101/46  110/65 | 6.4  9.7 | 90.5  66.3 | 154  159 | 1.53  1.48 | 185.5  183.5 | 14.0  14.3 | 339.4  280.7 | 179.0  262.8 | 78.2  83.0 |
|  | 3 | T0  T1 | 21 miles | A  A | 39.0  38.1 | 50  23 | 136  106 | 120/52  156/96 | >20.00  8.0 | 99.0  94.4 | 145  142 | 0.94  1.02 | 112.9  82.8 | 52.6  102.0 | 104.0  200.7 | 305.7  303.2 | 74.3  77.5 |
| II. | 5 | T0  T4 | >21 miles | P | >40.0  U/A | U/A  U/A | U/A  U/A | U/A  U/A | 26.8  7.0 | 38.6  45.8 | 124  100 | 1.16  0.75 | 136.1  >200.0 | 30.0  39.2 | 77.0  12.8 | 730.5  1220.1 | 56.3  42.6 |

**S1. Clinical and biochemical results for three EHI cases sampled within 30 minutes of incapacity (T0) and again 60 minutes (T1) or 4 hours (T4) following collapse.** Loc. 1. Treatment facility stationed at 14 mile-point on course (NB course design resulted in runners up to 21 miles being received here). Location II. Main medical tent stationed 100 m behind finishing line.
