# Supplementary material for: Relative changes in brain and kidney biomarkers with Exertional Heat Illness during a cool weather marathon
Source: PLoS One. 2022 Feb 17;17(2):e0263873. doi: 10.1371/journal.pone.0263873 (PMC8853487; doi:10.1371/journal.pone.0263873)
Supplement: S2 File — CK–creatine kinase; cysC–cystatin C; KIM-1 –Kidney Injury Molecule 1; NGAL–neutrophil gelatinase associated lipocalin; NSE–neuron specific enolase; sCr–serum creatinine; sNa–serum sodium; Ur–serum urea; TP–Total protein. (DOCX) [file pone.0263873.s002.docx]

|  | *Laboratory reference range* | **Baseline (B)** | **Completion (T0)** | P-value,  B vs T0 |
| --- | --- | --- | --- | --- |
| SBP  (mmHg) | - | 140 ± 20 | 117 ± 11 | <0.0001 |
| DBP  (mmHg) | - | 79 ± 12 | 67 ± 8 | <0.0001 |
| Heart rate  (b.min^-1^) | - | 62 ± 14 | 87 ± 14 | <0.0001 |
| Body mass*  kg | - | 72.30 ± 11.01 | 70.47 ± 10.81 | <0.0001 |
| NSE,  ug.L^-1^ | *1.85 – 4.14* | 3.17 [2.71,3.92] | 4.31 [3.47, 4.97] | <0.0001 |
| S100B,  ng.L^-1^ | *20 - 200* | 19.3 [13.43, 39.8] | 64.2 [43.2, 95.3] | <0.0001 |
| sCr  µmol.L^-1^ | *80 – 110 (men)*  *53 – 97 (women)* | 80 [64, 92] | 116 [91, 127] | <0.0001 |
| cysC  mg.L^-1^ | *0.55 - 1.00* | 0.86 [0.80, 0.93] | 1.10 [0.95, 1.21] | <0.0001 |
| NGAL  ug.L^-1^ | *42-177* | 50.7 [45.8, 60.9] | 132.2 [97.3, 162.9] | <0.0001 |
| KIM-1  ng.L^-1^ | *0 - 56.9* | 32.2 [21.9, 46.6] | 28.2 [18.1, 42.2] | <0.0001 |
| Copeptin pmol.L^-1^ | *1.0 -13.8* | 3.2 [2.5, 4.6] | 18.7 [7.1, 67.9] | <0.0001 |
| CK  IU.L^-1^ | *32 - 294 (men)*  *33-211 (women)* | 78.8 [57.4, 112.3] | 399.1 [229.2, 531.5] | <0.0001 |
| TP  g.L^-1^ | *57 - 82* | 71.6 [67.5, 73.6] | 71.1 [68.2, 74.1] | 0.79 |

**S2. Results for 30 successful finishers, at rested baseline B and upon marathon completion T0.** *CK – creatine kinase; cysC – cystatin C; KIM-1 – Kidney Injury Molecule 1; NGAL – neutrophil gelatinase associated lipocalin; NSE – neuron specific enolase; sCr – serum creatinine; sNa – serum sodium; Ur – serum urea; TP – Total protein.*
