# Supplementary material for: Relative changes in brain and kidney biomarkers with Exertional Heat Illness during a cool weather marathon
Source: PLoS One. 2022 Feb 17;17(2):e0263873. doi: 10.1371/journal.pone.0263873 (PMC8853487; doi:10.1371/journal.pone.0263873)
Supplement: S3 File — CK–creatine kinase; cysC–cystatin C; KIM-1 –Kidney Injury Molecule 1; NGAL–neutrophil gelatinase associated lipocalin; NSE–neuron specific enolase; sCr–serum creatinine; sNa–serum sodium; Ur–serum urea; TP–Total protein. (DOCX) [file pone.0263873.s003.docx]

|  | NSE  *ng.L^-1^* | S100b  *ng.L^-1^* | sCr  *µmol.L^-1^* | cysC  mg.L^-1^ | NGAL  *ug.L^-1^* | KIM-1  *ng.L^-1^* | Copeptin  *pmol.L^-1^* | CK  *IU.L^-1^* | TP  *g.L^-1^* |
| --- | --- | --- | --- | --- | --- | --- | --- | --- | --- |
| *B* | 4 | 20.44 | 66.1 | 1.01 | 47.98 | 51.28 | 2.028 | 61.2 | 72.6 |
| *T0* | 4.69 | 54.66 | 93.9 | 1.1 | 90.32 | 39.43 | 275.8 | 143.6 | 68.3 |
| *T24* | 3.29 | 39.83 | 76.7 | 0.95 | 59.96 | 55.26 | 6.34 | 285.8 | 62.4 |
| *B* | 3.71 | 31.16 | 61.8 | 0.84 | 65.86 | 15.37 | 5.053 | 86.9 | 65.4 |
| *T0* | 5.44 | 42.54 | 64.3 | 0.87 | 91.54 | 17.36 | 6.951 | 791.4 | 66.6 |
| *T24* | 3.68 | 36.78 | 64.5 | 0.88 | 108.7 | 36.4 | 5.243 | 1256.3 | 59.3 |
| *B* | 3.79 | 72.92 | 65.6 | 0.74 | 47.74 | 19.69 | 2.594 | 62.7 | 73 |
| *T0* | 3.63 | 59.27 | 95.3 | 0.95 | 166.86 | 19.8 | 9.071 | 970.5 | 72.5 |
| *T24* | 2.93 | 10.75 | 71.7 | 0.82 | 50.9 | 99.86 | 4.105 | 3010.3 | 70.2 |
| *B* | 2.55 | 24.73 | 57.1 | 0.76 | 52.5 | 40.83 | 2.783 | 169.2 | 73 |
| *T0* | 3.01 | 89.24 | 95.1 | 0.98 | 118.9 | 39.15 | 6.585 | 630.3 | 70.3 |
| *T24* | 2.58 | 33.99 | 52.3 | 0.76 | 60.96 | 52.08 | 2.959 | 677.2 | 64.9 |
| *B* | 2.57 | 25.51 | 121.2 | 0.92 | 44.32 | 53.98 | 5.384 | 1220.8 | 74.8 |
| *T0* | 4.39 | 103.52 | 128 | 0.95 | 56 | 65.74 | 23.561 | 2613.3 | 73.4 |
| *T24* | 2.68 | 69.12 | 114.9 | 0.95 | 51.18 | 73.89 | 12.444 | 4950.2 | 66.3 |
| *B* | 2.08 | 12.36 | 76.9 | 1.18 | 74.44 | 24.26 | 3.126 | 61.6 | 66 |
| *T0* | 3.38 | 72.66 | 98.4 | 1.43 | 118.94 | 22.13 | 19.92 | 365.2 | 67.2 |
| *T24* | 2.61 | 33.1 | 86.2 | 1.03 | 71.54 | 25.69 | 5.091 | 2081.1 | 60.1 |
| *B* | 2.32 | 49.99 | 77.3 | 0.94 | 46.36 | 20.8 | 1.853 | 62.6 | 76.7 |
| *T0* | 3.96 | 75.14 | 109.5 | 1.11 | 116.46 | 20.8 | 52.81 | 138.1 | 75.4 |
| *T24* | 2.89 | 29.65 | 75.3 | 0.9 | 71.88 | 9.78 | 1.781 | 347.8 | 69.5 |
| *B* | 2.31 | 22.85 | 57.3 | 0.88 | 54.28 | 18.41 | 3.19 | 72.5 | 66.5 |
| *T0* | 3.49 | 47.9 | 62.6 | 0.85 | 83.12 | 18.27 | 8.911 | 395.6 | 66.4 |
| *T24* | 2.88 | 16.49 | 55.6 | 0.93 | 56.78 | 16.4 | 3.539 | 1299.1 | 61.5 |
| *B* | 5.43 | 62.76 | 81 | 0.99 | 30.08 | 20.29 | 1.932 | 67.2 | 77 |
| *T0* | 4.89 | 103.52 | 101.5 | 1.06 | 198.92 | 7.42 | 66.65 | 369.2 | 75.5 |
| *T24* | 3.17 | 43.54 | 81.8 | 1 | 154.74 | 18.84 | 6.008 | 710 | 67.7 |
| *B* | 3.11 | 34.41 | 80.8 | 0.88 | 47.04 | 33.29 | 2.035 | 93.7 | 71 |
| *T0* | 4.47 | 56.41 | 108.1 | 1 | 121.28 | 29.39 | 223.2 | 370.7 | 67.9 |
| *T24* | 3.27 | 39.5 | 83.4 | 0.91 | 55.18 | 45.07 | 2.556 | 543.2 | 64.2 |
| *B* | 4.05 | 2.7 | 87.7 | 0.86 | 55.38 | 26.76 | 2.713 | 141.1 | 71.4 |
| *T0* | 3.96 | 26.9 | 116.9 | 1.06 | 104.72 | 28.58 | 63.07 | 408.2 | 75.7 |
| *T24* | 3.27 | 9.96 | 93.2 | 0.92 | 64.98 | 32.11 | 4.007 | 720.9 | 65.1 |
| *B* | 1.98 | 10.61 | 61.8 | 0.77 | 37.84 | 20.86 | 2.599 | 54.8 | 74.5 |
| *T0* | 4.03 | 70.97 | 137.5 | 1.35 | 130.06 | 21.98 | 110.2 | 195.4 | 81.2 |
| *T24* | 3.17 | 31.31 | 72 | 0.8 | 53.68 | 40.44 | 3.751 | 500.4 | 70.4 |
| *B* | 9.41 | 11.26 | 80.6 | 1.02 | 90.9 | 30.68 | 6.644 | 107.7 | 75.1 |
| *T0* | 4.19 | 84 | 116.3 | 1.42 | 162.44 | 27.37 | 6.294 | 141.9 | 72.8 |
| *T24* | 3.45 | 25.03 | 102.3 | 0.98 | 92.34 | 33.29 | 7.49 | 412.8 | 67 |
| *B* | 2.4 | 31.86 | 110.9 | 0.99 | 64.38 | 25.1 | 8.114 | 90.5 | 67.8 |
| *T0* | 3.13 | 78.15 | 158.3 | 1.42 | 151.02 | 26.67 | 38.63 | 278.2 | 72.3 |
| *T24* | 2.83 | 44.83 | 128.3 | 1.02 | 65.34 | 31.83 | 10.98 | 949.5 | 67.3 |
| *B* | 3.31 | 63.57 | 130.9 | 0.91 | 76.34 | 58.93 |  | 86 | 77.9 |
| *T0* | 4.04 | 137.02 | 125.9 | 1.03 | 110.22 | 62.28 |  | 1551.1 | 73.8 |
| *T24* | 3.36 | 88.62 | 119.3 | 1.05 | 91.7 | 64.47 |  | 11316.7 | 72.4 |
| *B* | 2.82 | 10.07 | 93.1 | 0.84 | 71.08 | 42.02 |  | 92.7 | 54.7 |
| *T0* | 3.52 | 40.5 | 211.2 | 1.81 | 200 | 44.44 |  | 379.9 | 69.5 |
| *T24* | 3.09 | 19.87 | 109.2 | 0.9 | 79.94 | 50.69 |  | 709.3 | 59.1 |
| *B* | 3.18 | 19.54 | 71.9 | 0.79 | 45.84 | 28.49 | 1.124 | 57.4 | 75.2 |
| *T0* | 3.18 | 35.62 | 89.7 | 0.98 | 97.44 | 23.73 | 15.72 | 129.6 | 72.8 |
| *T24* | 2.89 | 45.16 | 138.2 | 0.88 | 40.74 | 39.62 | 1.626 | 396.1 | 70.9 |
| *B* | 2.89 | 38.17 | 92.3 | 0.91 | 38.62 | 24.32 |  | 59.7 | 78.6 |
| *T0* | 3.8 | 67.49 | 100 | 0.94 | 72.84 | 23.23 |  | 124 | 71.4 |
| *T24* | 4.66 | 181.31 | 89.6 | 0.93 | 58.06 | 17.18 |  | 387.3 | 71.1 |

**S3. Individual biochemical results for 18 successful finishers (n=15 for copeptin), at rested baseline B, upon marathon completion T0 and next-day T24.** *CK – creatine kinase; cysC – cystatin C; KIM-1 – Kidney Injury Molecule 1; NGAL – neutrophil gelatinase associated lipocalin; NSE – neuron specific enolase; sCr – serum creatinine; sNa – serum sodium; Ur – serum urea; TP – Total protein.*
