# Supplementary material for: Relative changes in brain and kidney biomarkers with Exertional Heat Illness during a cool weather marathon
Source: PLoS One. 2022 Feb 17;17(2):e0263873. doi: 10.1371/journal.pone.0263873 (PMC8853487; doi:10.1371/journal.pone.0263873)
Supplement: Attachment — Submitted filename: Responses to second review of PONE-D-21-12312R1.docx [file pone.0263873.s005.docx]

Additional Editor Comments: *Reponse*s

1) If the authors answer the second reviewer adequately, I will accept the manuscript without another round of external reviews.

*Thank you, we have endeavoured to do so below.*
2) If possible, please provide information on the age of runners experiencing exertional heat illness. If not possible, the Academic Editor understands the unfavorable/unpredictable conditions of data collection.

*Thank you for your understanding, please see below.*
3) Please consider that the lack of information on the exercise-induced change in plasma volume (as determined from hematocrit and hemoglobin measurements) is a limitation of the present study. Please also consider carrying out these analyses in your future studies because they may be academically relevant.

*We shall, please see full response below.*
4) Please indicate the volume of blood samples.

*13 ml blood, please see below.*
5) The authors wrote the following information twice in the manuscript: “henceforth referred to as ‘successful finishers’” (lines 118 and 119, 140 and 141). The authors may want to delete the repeated information in lines 140 and 141.

*Thank you, we have done so.*
6) Line 144. Please consider replacing “mins” with “minutes”. It seems that the authors have not abbreviated this measuring unit throughout the manuscript.

*Thank you, we have done so.*
7) Line 222. Please consider replacing “14:00 and 15:00 PM” with “14:00 and 15:00” or “2:00 and 3:00 PM”.

*Thank you, we have done so.*
8) Please consider providing updated information for references 7 and 14.

*Thank you, we have done so.*
9) Please consider providing complete information for reference 29 (i.e., the volume, issue, and page numbers).

*We have done so for reference 28, which we assume you meant – apologies for this omission.*
10) Please consider reducing the excessive white space between panels in Figure 1.
11) Please consider removing a blank column in the supplementary Table 1.

*Thank you for spotting this, we have done so.*
12) The Academic Editor agrees with the maintenance of data regarding S100B.

*Thank you.*

Reviewer 2 Comments: *Reponse*s

The authors conclude that “The novel biomarker candidates for EHI outcomes in cool-weather endurance exercise, early elevations in NSE and copeptin provided sufficient discrimination to suggest utility at point-of-incapacity” however the two major points I indicate below should be answered, thus the conclusion of the article will be more honest.

First, it is essential to indicate more characteristics of the sample, especially those affected with EHI. For example, the interpretation of the data would change if all 8 volunteers were over 50 years old. The present reviewer does not understand how such simple information can be neglected in the study, as the authors themselves claimed to have had some kind of contact with the sample after they recovered: “For EHI cases who initially lacked mental capacity to consent for themselves ( AVPU grade V to U, or A with any concern for capacity) we proceeded with presumed consent until they were deemed able to give it retrospectively.” In addition to age, another variable as BMI would be of great importance.

*We have been unable to do so – the nature of a recreational event seems to favour participants who have been incapacitated wanting to move swiftly on with their day/conclusion to the unfortunate event of EHI supervening, hence conditions of data collection were suboptimal. We have accounted for this and the limitation below at lines 401-407.*

Second, with my question “How can the authors argue that the increase in certain blood variables occurred due to the reduction in plasma volume (because of the marathon and dehydration) and not due to real increases in the variables?” I was expecting the authors to argue whether they corrected the values of the biomarkers for variations in plasma volume (DILL, DB; COSTILL, DL Calculation of percentage changes in volumes of blood, plasma, and red cells in dehydration. J Appl Physiol, v. 37, No. 2, pp. 247-248, 1974). If this correction was not made, I suggest putting this limitation in the study.
*Please see lines 401-407.*

Lastly a minor question: Which was the volume of blood samples collected on the time-points?
These simple information requested would be of great relevance to the researchers in this field, as this is an article with unprecedented results.

*4.5 ml for plasma, 8.5 ml for serum = 13 ml, now documented in Methods section at lines 140 and 169.*
