# Supplementary material for: Relative changes in brain and kidney biomarkers with Exertional Heat Illness during a cool weather marathon
Source: PLoS One. 2022 Feb 17;17(2):e0263873. doi: 10.1371/journal.pone.0263873 (PMC8853487; doi:10.1371/journal.pone.0263873)
Supplement: Attachment — Submitted filename: 20211116 PLOS One reviewer comments response.docx [file pone.0263873.s004.docx]

5. Review Comments to the Author

Reviewer #1: Thank you for the opportunity to review the current manuscript. In this work, Stacey and colleagues have examined whether brain-enriched (NSE and S100B) and renal system-related (cysC, KIM-1, and NGAL) biomarkers and copeptin in blood can discriminate marathon runners suffering from EHI and successful marathon finishers without signs of EHI.

The subject of the current investigation is sound and clinically relevant. The literature of biochemical diagnostics of EHI among athletes is scarce. The group have conducted a study that is generally challenging to perform given the potentially methodological caveats such selection bias, incomplete sampling series and small sample size-related issues. It is worth keeping in mind that this study is the first of its kind and the results are therefore preliminary.

I enjoyed reading the paper. My suggestions for improving the manuscript are minor and relate mainly to describing the limitations of the methods, presenting the results, and putting the findings in context in a biochemical sense. Detailed comments below:

Introduction
- Previous literature has been described adequately.
- The clinical problem for which the study is designed is well described.
- The selection of AKI biomarkers seems to be appropriate but for based on the current literature (and also cited publication 16), the decision not to include more brain-specific biomarker GFAP is unfounded.

*Many thanks. We have now described, in our revised discussion, the reasons for not incorporating GFAP in this initial preliminary work. This is instead of introducing it as a relevant (but un-investigated, in this study) marker at the top of the document, so as not to detract from detail on the markers we do actually report.*

Methods
- The methods are appropriate overall.
- Studying brain damage with S100B that is known to rise in physical exercise and NSE that is affected by haemolysis without including e.g., GFAP and perhaps UCH-L1 is theoretically unsound. Of course, the strength of the methods is that NSE is a good choice because it is associated with systemic and neural inflammation (e.g., in SAE).

*Please see above comment on relative dearth of information for SAE prior to the execution of the study, with rationale updated in discussion and future suggestions now including GFAP.*

- The main limitation is description of the collapsed runners:

i) it is not described whether there were other collapsed runners with EHI in the event than those eight who were enrolled in the study (not possible to assess selection bias, age- and gender matching issues),

*Thank you for assisting us with these clarifying details, we have now categorically stated in the Results that the recruited cases represented 100% of EHI diagnoses diagnosed and initially treated at the on -course medical facilities, while acknowledging in the Discussion that casualties taken from point of collapse on the course direct to hospital may have been missed (though we were aware of no such transfers on this occasion).*

ii) the demographics of collapsed runners is not described, iii) it is not clear whether collapsed runners had a history of previous kidney conditions, traumatic brain injuries or other CNS conditions such tumours, migraine, stroke of CNS infections. Think that ii) and iii) would have been relatively easy to find out from the runners as they recovered.

*In practice, once collapsed runners were recovered sufficient to begin providing detailed collateral information on their personal particulars/circumstances, we found that they prioritised self-discharge from medical facilities. Several were visiting from out-of-area and had travel arrangements to honour, friends and family to reunite with, were keen to re-fuel and recover in their own way outside of the medical tents (which were busy with nonEHI presentations). Therefore the demographic data we have provided (gender) represent what was possible for our small teams to collect in the course of processing samples in austere conditions and we do not have retrospective access or general data protection authority to enter the stored clinical notes for more detail on these cases and any potential co-morbidities.*

Results
- The results section reads well.
- The authors address the decrease of NSE levels in two collapsed runners in the discussion section, but the data is only available in the Supplementary Table 1 and not mentioned in the body text.

*Now addressed in main body text, thank you.*

- Supplementary Table 1 is labelled incorrectly in the supplementary file, please correct.

*Corrected in line with preferred journal formatting, thank you.*

Discussion
- The authors put the current findings into context well.
- On the line 320, please correct “radiographically-demonstrable traumatic injury” to “radiographically-demonstrable traumatic intracranial injury”

*We have done so, thank you*

- In patients with traumatic brain injury, the extent of extracranial confounding in polytrauma patients with TBI has been discussed, but studies suggest that extracranial leak of S100B is quickly eliminated (Savola O, J Trauma. 2004 Jun; 56(6):1229-34; and da Rocha AB. Clin Chem Lab Med. 2006; 44(10):1234-42.). Given the sampling time points are very close to each other, I find very problematic to discuss the S100B levels in collapsed runners.

*Unfortunately we could not find supporting data on rates of in vivo S100B decay in the paper by Savalo et al (kindly referenced by the reviewer), but did note that sampling was completed within 6 hours in the various degrees of injury assessed and showed discrimination for brain injury and large extracranial injury versus smaller extracranial injuries and control subjects. In the other paper suggested, by da Rocha et al, critically brain-injured patients were sampled at study entry T0 (median 10.9 h post injury), T+1 day and T+7 days, showing significant relative elevations for T0 vs T24 and between survivors/non survivors at T24. While we acknowledge that, for good practical reasons, studies recruiting brain injured patients from trauma are limited in their ability to assay S100B at close to point-of-incapacity as we were able to do for EHI cases in the marathon – and this leaves a relative gap in that literature – data do exist to demonstrate cellular release of S100B as early as 3 hours post-injury in vitro. Though limited to 3 cases with data beyond the initial 30 minute period (T0) following incapacitation, our S1 supplementary table does show mild to moderate reductions in S100B at 1 hour post-insult (T1) – possibly compatible with correction of physiological haemoconcentration post-run and fluid resuscitation/recovery – but also, importantly, a relative rise at 4 hours in the one EHI case unwell enough to be admitted from the course facilities to the local hospital. Therefore if our study had been able to recruit and retain greater numbers of EHI cases with relatively greater severity of illness/injury over serial sampling points, as we intended, S100B may have presented greater interest/potential utility, especially at this later timepoint. As things stand, we feel that the S100B data nicely complement the (more neurally-specific) NSE data and that novelty remains in this comparison overall. Nevertheless, if the Editor feels strongly that S100B should be removed from the manuscript for publication, we would strongly consider this.*

- The possible demographic differences and also and possible poorer physical performance of collapsed runners compared to successful finishers is a major confounding factor as it is likely has partly exposed them to EHI (reflected in also the biomarker levels). This should be acknowledged.

*Respectfully, we tend to see things the other way, and would prefer to acknowledge the possibility that runners who experienced EHI were more likely to be highly motivated, as has been reported in post-EHI interviewing of military cases (ABRIAT, A., BROSSET, C., BRÉGIGEON, M. & SAGUI, E. 2014. Report of 182 cases of exertional heatstroke in the French Armed Forces. Military medicine, 179, 309-314), thus perhaps ‘fit enough’ to drive themselves to the point of collapse. On balance, we would like to acknowledge the need for a future study with better potential to characterise collapsed cases, including in-race monitoring/surveillance (as can be achieved with the chips issued with running numbers as an increasingly ubiquitous standard for runners to log and retrospectively review their time ‘splits’ across the distance) and post-event questionnaires to including training volumes, predicted race times etc, as well as a bigger team to capture the more basic demographic data highlighted above.*

In summary, this is a well-conducted preliminary study with interesting findings. I am looking forward to seeing the next version of the manuscript.

*We are very grateful for your time, input and expertise, thank you.*

Reviewer #2: Stacey and colleagues aimed primarily to determine whether some markers could effectively discriminate marathon runners affected by EHI from successful finishers completing the event on the same day. Secondary aims were to examine changes in these biomarkers over time and to relate indirect evidence for organ injury to likely precipitants, co-factors or other indicators relevant to the episodes of collapse observed.
The results are quite relevant. The manuscript is written in a well-organized fashion and has an appropriate language.
Considering that PLOS ONE objectively concentrates on the technical aspects of a study rather than the more subjective evaluations, the presented paper can be published in this journal. However, there are some important questions in the methods to be clarified and corrected.


- The authors say that the time-point of blood collection in the successful finishers and collapsed runners was “as feasible”. It is important you present the real time-point means and standard deviations.

*We agree that real time-pint means would be ideal. However the conditions prevailing in this field based study of mass participation exercise - with emergency responders recovering collapsed casualties from the course to the appropriate medical facilities and conducting clinical handover under pressure of time in busy medical tents – meant that in a number of the casualties timings were provided as 10 or 20 minute integers, with sampling performed at a similarly approximate time point, but confirmed as no more than 30 minutes from collapse. Even the intensive care environments from which the neuro-biomarkers have been reported in brain injury and sepsis-associated encephalopathy are relatively controlled, but studies often default to the nearest whole hour post-injury in reporting. We have previously published field work in which copeptin was sampled within 20 minutes of exertion ceasing (Stacey MJ, Delves SK, Britland SE,et al. Eur J Appl Physiol. 2018;118:75-84.) and deemed 30 minutes to be acceptable in this instance, given the added complications of marathon logistics and, ultimately, the practical question we were attempting to address of how these markers might identify/discriminate EHI. We feel that the data support this approach, with high discriminant value shown by both NSE and copeptin.*

- How was the core temperature measurement? Please insert this information in the methods section.

*In the Methods section on collapsed runners, we have already stated* ‘core body temperature measured rectally (Intellivue integrated thermistor, Philips Healthcare, Amsterdam, Netherlands).’ *Please do let us know if further clarification is required.*

- Please insert the collapsed group age in table 1 or the text.

*Please see comments above about the logistical constraints that limited the fuller data collection that would otherwise have been ideal in this situation. While we do have date of birth data for the casualties treated at Medical Facility I, Medical Facility II (finish-line) was dealing with high volumes of non-EHI (including hypothermia, myocardial infarction and post-exercise collapse, some of which was precipitated by the relatively inclement conditions) and introduced limitations to data collection beyond confirming the EHI diagnosis has been assigned clinically, the gender of the patient and their informed consent to participate in the study. A larger team may have improved the possibility of adding further casualty data, however we were necessarily split across two sites which limited availability of personnel and was in fact necessary given that half of the cases presented at Medical Facility I.*

- How can the authors argue that the increase in certain blood variables occurred due to the reduction in plasma volume (because of the marathon and dehydration) and not due to real increases in the variables?

*We apologise if we have not been clear here – in the statement on copeptin, the stable surrogate for arginine vasopressin, we have now noted that ‘both hypovolaemia and hypertonicity being potent, classical stimuli to the release of AVP/copeptin.’*

- Please insert the relative humidity data and time of the marathon.

*We have now inserted the hours of the event, as suggested, in relation to relevant meteorological conditions in the first line of the Results. We do not have access to raw relative humidity data, however, but do have WBGT recordings for the hours in which the marathon was conducted. However our understanding is that relative humidity and wet bulb measures have little relevance to thermoregulation in marathon conditions when the ambient temperature is as low as we report for the study. Therefore WBGT has been omitted; we could provide this as a supplementary data table, but have been informed previously in relation to another study that this would be nonsensical at low ambient temperature.*

.
- Page 3, line 59, Introduction: The phrase “and can be greater in shorter-distance events” is referenced as number 8. However, the cited article is about “Heat stroke risk for open-water swimmers during long-distance events” and does not support this affirmation.

*Thank you, our apologies, for brevity we have removed the statement and the duplicate reference (number 9 appearing as both 8 and 9, our error) is now singular and appropriately positioned.*

Reviewer #3: The manuscript etiteled „Relative changes in brain and kidney
biomarkers with Exertional Heat Illness during a cool weather marathon“
was to investigate whether biomarker surrogates for end-organ damage
sampled at point-of-care could discriminate EHI versus successful
marathon performance“ should be accepted in this version because:
-Abstract provided the profile of the manuscript.
-The study gained ethical approval.
-The methods are clear and replicable.
-All the results presented match the methods described and the
statistical analysis appropriate to the research question and study
design.
-The data are presented clearly and appropriately.
-The paper uses appropriate references in the correct style to promote
understanding of the content.

*Thank you.*
